# Antihypertensive Effects of the *Vitex cienkowskii* (Verbenaceae) Stem-Bark Extract on L-NAME-Induced Hypertensive Rats

**DOI:** 10.1155/2021/6668919

**Published:** 2021-03-05

**Authors:** Mireille Flaure Metchi Donfack, Albert Donatien Atsamo, Roméo Joël Temdié Guemmogne, Omer Bébé Ngouateu Kenfack, Alain Bertrand Dongmo, Théophile Dimo

**Affiliations:** ^1^Department of Animal Biology and Physiology, Faculty of Science, University of Yaoundé I, P.O. Box 812, Yaoundé, Cameroon; ^2^Department of Biological Sciences, Faculty of Science, University of Ngaoundéré, P.O. Box 454, Ngaoundéré, Cameroon; ^3^Department of Animal Biology, Faculty of Science, University of Douala, P.O. Box 24157, Douala, Cameroon

## Abstract

*Vitex cienkowskii* stem-bark is used in Cameroonian traditional medicine to treat cardiovascular diseases including hypertension. In previous studies, the methanol/methylene chloride stem-bark extract of *Vitex cienkowskii* (MMVC) showed a preventive activity in L-NAME-induced hypertension and improved blood pressure of spontaneously hypertensive rats. The present study investigated the curative effects in L-NAME-induced hypertensive rats (LNHR). Hypertension was induced in rats by oral administration of L-NAME (40 mg/kg/day) for 28 days. The animals were divided into 2 groups: one group of 5 rats receiving distilled water (10 ml/kg) and another 20 rats receiving L-NAME. At the end of 4 weeks of administration of L-NAME, the animals were divided into 4 groups of 5 rats each: one group of hypertensive rats receiving distilled water, another one receiving captopril (25 mg/kg), and two groups of hypertensive rats receiving MMVC at doses of 200 and 400 mg/kg, respectively. Body weight, food, and water intake were measured weekly. At the end of the treatment, blood pressure and heart rate were recorded by invasive method. Whole heart, left ventricle, kidneys, and liver were weighed. The effects of plant extract on lipid profile and oxidative stress markers, as well as markers of hepatic and renal functions were assessed spectrophotometrically according to well described protocols. Results show that L-NAME significantly increases the mean arterial blood pressure (MABP), atherogenic index, lipid profile, and creatinine and transaminase activities of normotensive rats. MMVC significantly reduced the blood pressure in LNHR. Body weight, food and water intake, left ventricular hypertrophy, antioxidant level, renal and hepatic markers, and lipid profile were improved by the treatment with MMVC. The curative effect of MMVC on L-NAME-induced hypertension is probably related to its antihypertensive, hypolipidemic, and antioxidant properties. These results confirmed the use of *Vitex cienkowskii* for the treatment of hypertension in traditional medicine.

## 1. Introduction

Hypertension is one of the most prevalent causes of cardiovascular diseases. The development of an impaired vascular relaxation process is mainly due to endothelial dysfunction and oxidative stress [1]. As hypertension remains silent, it greatly increases the risk of complications. The ideal treatment of hypertension must lead to a protection from tissue damages mainly the heart, vessels, brain, and kidneys through the management of blood pressure. Therefore, drug development strategies are focused on discovering and designing molecules that could improve on this pathophysiological condition by acting on targets that regulate vascular smooth muscle contraction and relaxation pathways [2, 3]. Although nitric oxide (NO) plays an important role in endothelium-dependent vasodilatation, it is important to underline that NO is also involved in cardiac function as it has been reported [4]. Inhibition or chronic defect of NO leads to the development of arterial hypertension [5]. A significant drop in NO induced by administration of the nitric oxide synthesis inhibitor, L-NAME, promotes severe and progressive physiopathological injury consisting in the development of oxidative stress and structural organ injury [6]. In fact, the increase in vasculature stretch raises the NAD(P)H oxidase activity of membrane-bound wall of vessel, resulting in production of superoxide anion (O2^−^), the first product of this oxidative chain [7]. Oxidative stress occurs with the imbalance between reactive oxygen species (ROS) and antioxidant defense systems. The superoxide anion (O2^−^) reacts with NO and decreases its bioavailability, resulting in endothelial dysfunction and impairing vascular reactivity. This could lead to a decrease in microvascular perfusion and damages to organs such as the liver and kidneys [8]. In addition, the chronic inhibition of NOS induces hypertension with concomitant myocardial hypertrophy as a compensatory response in order to adapt the heart function facing a surcharge of pressure or volume [9]. The premature death due to severe damages in organs could be prevented by clinical treatment and control of elevated blood pressure [10]. Globally, more than 80% of the community rely on herbal remedies to treat various human diseases including arterial hypertension [11]. Interestingly, the World Health Organisation (WHO) has encouraged this alternative medicine for the prevention and treatment of diseases [12].

Activities of many plant extracts provide strong evidence that they might contain additional unidentified compounds contributing to the treatment of human diseases. *Vitex cienkowskii* is a plant of the family of Verbenaceae, which is commonly used in the Cameroonian traditional medicine for the treatment of cardiovascular diseases including hypertension. In previous studies, chemical investigations on the methanol/methylene chloride stem-bark extract of *Vitex cienkowskii* allowed the isolation of the known triterpenoids (oleanoic acid, salvin A, maslinic acid, *β*-sitosterol, and *β*-sitostérol-glycoside), a ceramide (tanacetamide), flavonoids, a 20-hydroxyecdysone, and a tetra-acetylajugasterone C, responsible for vasorelaxant activity and hypotensive effect [13, 14]. The concomitant administration of that plant extract with L-NAME prevented the development of hypertension in rats, by improving on the endothelial function and the oxidative stress status [15]. The aim of the present study was to investigate the curative effects in L-NAME-induced hypertensive Wistar rats (LNHR) with associated hypertension features such as oxidative stress, dyslipidemia, and organs injuries.

## 2. Materials and Methods

### 2.1. Preparation of the Plant Extract

*Vitex cienkowskii* fresh bark was collected in Foumban (West Region, Cameroon) in May 2016. The plant was identified at the National Herbarium (HNC) of Cameroon, in comparison with the existing voucher's specimen, deposited under number 32721HNC. The stem-bark was dried at room temperature and made into powder with a motor-driven grinder. The powder (1300 g) was macerated at room temperature (about 27°C) in 4 L of methylene chloride/methanol mixture (1 : 1) for 72 h and filtered. The filtrate was concentrated in a rotary evaporator at 70°C under reduced pressure and 38 g of a brown residue was obtained (W/W yield: 2.92%).

### 2.2. Experimental Animals

For this study, male Wistar rats aged 10–12 weeks (180–200 g) were used. The rats were housed in plastic cages and maintained in the animal house of the Department of Animal Biology and Physiology of the Faculty of Science, University of Yaounde I, Cameroon. They were kept under standard laboratory conditions with natural luminosity cycle, with free access to normal commercial diet and tap water *ad libitum.* Experimental protocols used in this study were performed according to the standard ethical guidelines for laboratory animal use and care, as described in the European Community guidelines, EEC Directive 86/609/EEC.

### 2.3. L-NAME-Induced Hypertensive Rats

A total of 25 normotensive Wistar male rats were randomly divided into two groups: the first group (vehicle, 5 rats) received distilled water (1 mL/100 g of body weight), and the second group (L-NAME, 20 rats) was treated with L-NAME (40 mg/kg) during 28 days. On the 29^th^ day, the rats of the second group were divided into four groups of 5 rats each which received, respectively, L-NAME and distilled water (1 mL/100 g of body weight) for the negative control, L-NAME and captopril (25 mg/kg) for the positive control, L-NAME and plant extract (200 mg/kg), or L-NAME and plant extract (400 mg/kg). All these products were dissolved in distilled water and given daily *per os* to the animals for a period of 28 days. During this period, food, water consumption, and body weight were recorded once a week. At the end of the treatment, blood pressure and heart rate of each animal were recorded.

### 2.4. Blood Pressure and Heart Rate Measurements

Blood pressure (BP) and heart rate (HR) of LNHR were recorded by invasive method under anesthesia (1.5 g/kg intraperitoneal injection of urethane) according to a method previously described [16]. The trachea was exposed and intubated to facilitate spontaneous respiration. A polyethylene catheter was inserted into the rat carotid artery and connected to a pressure transducer recording system (Biopac Student Lab MP35) for BP and HR measurement after thirty minutes period of equilibration.

### 2.5. Collection of Samples and Estimation of Biochemical Parameters

After the recording of hemodynamic parameters under anesthesia, blood samples were collected from carotids in dry tubes and allowed to stand for 30 minutes at room temperature. Serum obtained by centrifugation (3000 g for 10 min) was stored at −20°C. The heart, aorta, liver, and kidneys were removed rapidly, cleaned from adipose tissues, and weighed. The left ventricle plus septum and right ventricle were separated from the total heart and weighed. The whole heart and aorta were crushed in Mac Even solution while the liver and kidneys were crushed in Tris-HCl (50 mM) buffer to prepare 20% homogenates. After centrifugation (3000 g for 30 min), the supernatant was collected and stored at −20°C for biochemical analyses. Nitric oxide (NO) assay was performed using the Griess method [17], superoxide dismutase (SOD) activity was measured using the method of Misra and Fridovich [18], reduced glutathione (GSH) was evaluated using the method of Ellman [19], catalase was determined according to Sinha's method [20], and Malondialdehyde (MDA) concentration was determined using the procedure of Wilbur et al. [21]. Triglycerides, total bilirubin, total cholesterol, HDL-cholesterol levels, creatinine, ALAT, and ASAT activities were estimated by using specific colorimetric kits (Fortress Diagnostics). LDL-cholesterol was calculated according to Friedewald formula [22]. The atherogenic index (AI) was calculated following the formula used by Youmbissi et al. [23]. The markers of oxidative stress were evaluated in the homogenates of the heart, aorta, liver, and kidneys. The lipids were quantified in the serum. Creatinine was assessed in the kidneys and serum. Bilirubin, ASAT, and ALAT were assayed in the liver and serum. All these parameters were determined by measurement of the optical density of the reaction products at the corresponding wavelengths with spectrophotometer (Thermo Scientific Multiskan FC).

### 2.6. Statistical Analysis

The results were expressed as means ± SEM. The difference between the groups was compared using one-way analysis of variance (ANOVA) followed by Dunnett's test. *P* values less than 0.05 were considered significant. All analyses were performed using GraphPad Prism Software version 5.03.

## 3. Results

### 3.1. In Vivo Effects of MMVC in L-NAME-Induced Hypertensive Rats

#### 3.1.1. Blood Pressure and Heart Rate

**Figure 1:**
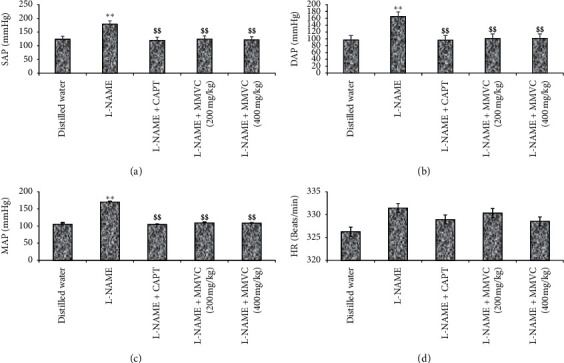
Effects of the methanol/methylene chloride extract of *V. cienkowskii* on arterial pressure and heart rate. Each bar represents the mean ± SEM; *n* = 5; ^*∗∗*^*P* < 0.01 significant difference compared to control (rats receiving distilled water); ^$$^*P* < 0.01 significant difference compared to hypertensive control (rats receiving L-NAME only); L-NAME + CAPT: rat treated with L-NAME and captopril at the dose of 25 mg/kg; L-NAME + MMVC (200 mg/kg): rats treated with L-NAME and plant extract (MMVC) at the dose of 200 mg/kg; L-NAME + MMVC (400 mg/kg): rats treated with L-NAME and plant extract (MMVC) at the dose of 400 mg/kg; SAP: systolic arterial pressure (a); DAP: diastolic arterial pressure (b); MAP: mean arterial pressure (c); HR: heart rate (d).

The values of systolic arterial pressure (SAP) (Figure 1(a)), diastolic arterial pressure (DAP) (Figure 1(b)), mean arterial pressure (MAP) (Figure 1(c)), and heart rate (HR) (Figure 1(d)) for the different groups at the end of treatment are presented in Figure 1. The treatment of rats with L-NAME increased significantly (*P* < 0.01) the systolic, the diastolic, and the mean arterial pressure when compared to rats receiving distilled water. For example, MAP increased from 105.44 ± 2.34 mm Hg in the control group to 169.97 ± 5.62 mm Hg in the negative control. Captopril (25 mg/kg) and MMVC (200 and 400 mg/kg) significantly (*P* < 0.01) reduced the MAP by 41.23%, 38.04%, and 35.92%, respectively (Figure 1(c)). The heart rate increased in nonsignificant manner (*P* > 0.05) in the groups that received L-NAME (40 mg/kg) when compared to the control group (vehicle). Captopril or MMVC did not induce a significant variation of the HR (Figure 1(d)).

#### 3.1.2. Body Weight

**Figure 2:**
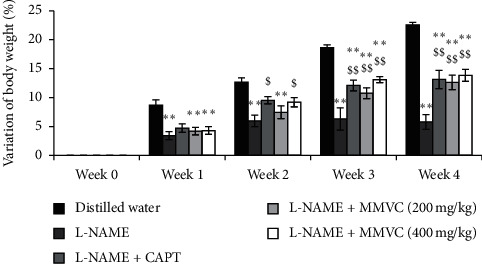
Effects of the methanol/methylene chloride extract of *V. cienkowskii* on body weight. Each bar represents the mean ± SEM; *n* = 5; ^*∗*^*P* < 0.05; ^*∗∗*^*P* < 0.01 significant differences compared to control (rats receiving distilled water); ^$^*P* < 0.05; ^$$^*P* < 0.01 significant differences compared to hypertensive control (rats receiving L-NAME only); L-NAME + CAPT: rats treated with L-NAME and captopril at the dose of 25 mg/kg; L-NAME + MMVC (200 mg/kg): rats treated with L-NAME and plant extract (MMVC) at the dose of 200 mg/kg; L-NAME + MMVC (400 mg/kg): rats treated with L-NAME and plant extract (MMVC) at the dose of 400 mg/kg; Week 0: week before the 1^st^ week of treatment; Week 1–Week 4: the 1^st^ to 4^th^ week of treatment.

The body weight decreased in significant manner (*P* < 0.01) during the experimental period, in the negative control animals, when compared to the normal control group. Treatment of LNHR with MMVC (200 and 400 mg/kg) or captopril (25 mg/kg) significantly (*P* < 0.01) improved the body weight. Thus, the percentage weight gain of rats of the negative control at the end of the fourth week of treatment with L-NAME was 4.79%, whereas those of groups treated with captopril and MMVC at the doses of 200 and 400 mg/kg were 13.12%, 12.84%, and 13.84%, respectively (Figure 2).

#### 3.1.3. Food and Water Intake

**Figure 3:**
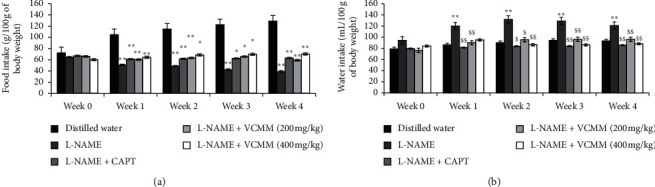
Effects of the methanol/methylene chloride extract of *V. cienkowskii* on food (a) and water intake (b). Each bar represents the mean ± SEM; *n* = 5; ^*∗*^*P* < 0.05; ^*∗∗*^*P* < 0.01 significant differences compared to control (rats receiving distilled water); ^$$^*P* < 0.01 significant difference compared to hypertensive control (rats receiving L-NAME only); L-NAME + CAPT: rats treated with L-NAME and captopril at the dose of 25 mg/kg; L-NAME + MMVC (200 mg/kg): rats treated with L-NAME and plant extract (MMVC) at the dose of 200 mg/kg; L-NAME + MMVC (400 mg/kg): rats treated with L-NAME and plant extract (MMVC) at the dose of 400 mg/kg; g or mL/100 g of body weight: quantity consumed in g or mL per week per 100 g of body weight; Week 0: week before the 1^st^ week of treatment; Week 1–Week 4: the 1^st^ to 4^th^ week of treatment.

As shown in Figure 3, food consumption of all test groups significantly decreased during the experimental period compared to the normal control group. At the end of the fourth week, the percentage decrease values stood, respectively, as 69.81%, 50.97%, 53.97%, and 45.44%, respectively, in the negative control, captopril-treated group, and rats treated with the MMVC at the doses of 200 and 400 mg/kg. Water intake increased significantly (*P* < 0.01) during the four weeks of treatment in LNHR compared to the normal control. Captopril and MMVC (200 and 400 mg/kg) significantly blunt (*P* < 0.01) the thirst registered in LNHR from the first week (Figure 3(a)). The water intake of LNHR that was 132.00 mL/100 g of body weight at the end of the first week dropped to 83.63, 95.44, and 86.44 mL/100 g of body weight, respectively, in rats treated with captopril and MMVC 200 and 400 mg/kg (Figure 3(b)).

#### 3.1.4. Relative Weight of Organs

The effects of the plant extract on the relative weight of organs of LNHR following a 28-day treatment period are summarized in Table 1. A significant (*P* < 0.05) increase in relative weight of the whole heart (15.06%), the left ventricle (19.87%), and the liver (30.51%) was observed in LNHR compared to the control. Neither captopril nor MMVC at doses of 200 and 400 mg/kg induced an increase in relative weight of the whole heart and liver observed in LNHR. However, treatment with captopril or MMVC (200 and 400 mg/kg) during four weeks improved on the left ventricular hypertrophy observed in nontreated LNHR by reducing its percentage from 14.15, 10.28, and 10.73% compared to the hypertensive control, respectively (Table 2).

#### 3.1.5. Hepatic and Renal Function Biomarkers

The effects of four-week treatment with MMVC on hepatic and renal function of LNHR are presented in Table 2. Hepatic and blood bilirubin did not show any significant difference in either nontreated LNHR or control rats. MMVC at the dose of 400 mg/kg significantly (*P* < 0.01) reduced the hepatic and blood bilirubin levels by 29.15 and 11.85%, respectively, compared to the control.

Subchronic treatment of animals with L-NAME significantly (*P* < 0.01) increased the blood and hepatic levels of ASAT and ALAT compared to the control animals. Captopril and MMVC at the doses of 200 and 400 mg/kg significantly (*P* < 0.01) reduced the blood ASAT levels (36.77, 48.09, and 39.05 %, respectively) and ALAT levels (32.43, 30.79, and 31.06, respectively). Captopril and MMVC at the doses of 200 and 400 mg/kg significantly (*P* < 0.01) decreased the hepatic ALAT levels in hypertensive rats to 19.18, 21.21, and 21.33%, respectively, while the hepatic ASAT level was reduced to 39.05% by MMVC at the dose of 400 mg/kg.

Blood and renal creatinine levels significantly (*P* < 0.01) increased by 81.13% and 91.46%, respectively, in experimental LNHR compared to the control animals. Treatment of hypertensive rats with captopril and MMVC at the dose of 400 mg/kg caused a significant (*P* < 0.05) decrease in the rise of creatinine to 24.48 and 28.13% in the serum and to 44.59 and 51.59% in the kidneys compared to the hypertensive control animals. MMVC at the dose of 200 mg/kg significantly (*P* < 0.05) decreased creatinine by 45.22% in the serum.

#### 3.1.6. Lipid Profile

Table 3 summarizes the effects of MMVC on lipid profile of LNHR. A significant increase in total cholesterol (42.49%; *P* < 0.01), LDL-cholesterol (23.12%; *P* < 0.05), and triglycerides (49.91%; *P* < 0.05) and a decrease in HDL-cholesterol (156.06%; *P* < 0.01) were registered in LNHR compared to the normal control animals. MMVC at the dose of 400 mg/kg significantly (*P* < 0.01) lowered the rise in total cholesterol (42.83%), triglycerides (52.40%), and LDL-cholesterol (73.93%) and reduced HDL-cholesterol (86.15%) observed in nontreated hypertensive rats. Captopril reduced triglycerides (39.08%; *P* < 0.01) and increased HDL-cholesterol (80.00%; *P* < 0.01) compared to LNHR.

As depicted in Table 3, when compared to the normal control group, L-NAME administration induced a significant (*P* < 0.001) 5.27-fold elevation of atherogenic index (0.81 ± 0.17 to 4.27 ± 0.57). However, when these animals were concomitantly treated with captopril (25 mg/kg) along with L-NAME, a 2.32-fold significant (*P* < 0.001) decrease of the atherogenic index was noted. In groups treated concomitantly with L-NAME and MMVC at the doses of 200 and 400 mg/kg, a 3.95-fold and a 7.23-fold decrease of the AI were, respectively, observed.

#### 3.1.7. Oxidative Stress

As shown in Table 4, rats treated with L-NAME during 8 weeks showed a significant improvement on antioxidant parameters in some selected organs (the aorta, heart, liver, and kidneys) tested compared to the control group. Treatment during four weeks with captopril significantly (*P* < 0.01) raised NO levels to 108.54, 89.32, 45.65, and 35.77%, respectively, in the aorta, heart, liver, and kidneys compared to the hypertensive control group. MMVC at the doses of 200 and 400 mg/kg significantly (*P* < 0.01) increased the level of NO compared to the hypertensive control animals. This increase reached a maximum percentage of 95.45% (aorta) at the dose of 400 mg/kg.

The level of GSH was significantly (*P* < 0.01) reduced in the aorta (21.15%), heart (20.09%), liver (18.59%), and kidneys (14.91%) of the LNHR compared to the control. Comparative to the hypertensive control, treatment with captopril and MMVC (200 and 400 mg/kg) significantly (*P* < 0.01) increased the concentration of GSH in these different organs (Table 4).

Four-week treatment of rats with L-NAME induced a significant (*P* < 0.05-*P* < 0.01) increase in the concentration of MDA by 118.88% in the aorta, 50.80% in the heart, 45.98% in the liver, and 76.27% as compared to the control. Treatment of hypertensive rats with captopril and MMVC at the doses of 200 and 400 mg/kg significantly (*P* < 0.01) reduced the level of MDA in the aorta by 75.02% (MMVC, 400 mg/kg) and in the heart by 25.95% (MMVC, 400 mg/kg). A significant (*P* < 0.05) decrease in the activity of SOD and catalase was also observed in LNHR compared to the control animals. Captopril and MMVC significantly (*P* < 0.05) increased the activities of these enzymes when compared to the hypertensive control group, reaching 168% and 100% at the doses of 200 mg/kg and 400 mg/kg, respectively, in the aorta (Table 4). MMVC at the doses of 200 and 400 mg/kg reduced the catalase activity depletion observed in the aorta and heart of hypertensive rats. The percentage decreases in the aorta and heart were, respectively, 86.75% and 87.97% at the dose of 200 mg/kg (MMVC) and 104.60% and 113.44% at the dose of 400 mg/kg (MMVC). Likewise, treatment of rats with captopril increased the catalase activities in the heart (92.02%; *P* < 0.05) and in the liver (147.09%; *P* < 0.01).

## 4. Discussion

*Vitex cienkowskii* is a plant commonly used in Cameroon for the treatment of cardiovascular disorders [13]. Previous studies showed that the methanol/methylene chloride extract of *Vitex cienkowskii* (MMVC) would be able to prevent hypertension in L-NAME-induced hypertensive rats (LNHR). The present work investigated the therapeutic effects of MMVC in LNHR.

The inhibition or chronic deficit of nitric oxide (NO) in the body is well known to induce severe diseases such as arterial hypertension [5]. Experimental arterial hypertension induced by L-NAME is marked by a reduction of NO/GMPc activity, activation of renin angiotensin aldosterone system (RAAS), and increase of sympathetic activity leading to an increase in vascular peripheral resistance resulting in a rise in blood pressure [24]. In this study, the chronic administration of L-NAME to normotensive rats induced a significant increase of mean arterial blood pressure (MAP). Same results have already been obtained by many researchers [25, 26]. MMVC administered at the doses of 200 and 400 mg/kg decreased the high blood pressure recorded in LNHR. It has been reported that NO may be involved in the *V. cienkowskii* extract-mediated vasorelaxation, with a significant role of the NO-cGMP system [13]. Thus, the antihypertensive effect of MMVC could be due to its vasorelaxant activity through the increase in NO release after the subchronic administration of the plant extract. These findings are in accordance with our previous works which showed that MMVC administrated simultaneously with L-NAME prevented the development of arterial hypertension [15]. Captopril, an inhibitor of angiotensin conversion enzyme, rectified the hypertension observed by increasing NO synthase activity and by inhibiting the production of angiotensin II. Moreover, these mechanisms act by the depletion of salts and water reabsorption, resulting in reduction of blood pressure [27, 28] without modifying the heart rate of LNHR.

In addition to its effects on arterial pressure, many studies showed that NO is involved in the regulation of food and water consumption [23, 24]. The current study showed an improvement on body weight related to increase in food intake and a reduction of thirst in hypertensive rats treated with MMVC. This result could be explained by the increase of NO in hypertensive rats after treatment with MMVC. It has also been reported that a rise in NO is able to increase food intake by regulating the appetite center in the brain [29, 30] and decrease of angiotensin II [31], leading respectively to elevation of food intake and reduction of water intake.

Arterial hypertension is a main risk factor responsible for serious tissue damages characterized by endothelial dysfunction [32], cardiac alterations [9, 33], and alteration of hepatic and renal functions [9, 32]. In this work, MMVC prevents the left ventricular hypertrophy observed in LNHR. In this model, ventricular hypertrophy is closely related to fibrosis and cardiomyocytes remodeling due to permanent high pressure associated with low concentration of NO [34]. Ventricular hypertrophy is a myocardial adaptation mechanism in response to chronic overloading in pressure or volume, leading to overproduction of angiotensin II. Then, angiotensin II induces cardiomyocytes growth resulting in an increase in ventricular weight [35, 36]. These observations suggest that the effect of treatment of ventricular hypertrophy with MMVC might be due to reduction of blood pressure consecutive to the rise in NO. The kidneys play an important role in the balance of water and electrolytes in the body. Hypertension is also a major risk factor that predisposes the liver and kidney to disorders [37, 38]. The chronic inhibition of nitric oxide induces microvascular changes which lead to perturbations in hepatic and renal perfusion resulting in severe damages in the organs [39]. In the current study, MMVC reduced the rise of blood and renal creatinine levels observed in LNHR. The elevation of renal NO registered after treatment with MMVC would be responsible for the improvement of the creatinine level in LNHR. Hepatic circulation is peculiar because more than 90% of the body blood flows through the hepatic portal vein. Hypertension induces microvascular changes. The nonsufficient perfusion of liver, which can be observed in several models of hypertension, is marked by an increase in hepatic enzyme activities [8, 40]. MMVC corrected the rise of liver weight, as well as the increase in bilirubin level and ASAT and ALAT activities observed in LNHR. This effect could be related to the increase in hepatic NO observed in this study [8], which improves hepatic microcirculation leading to improvement of hepatic enzyme activities and liver functions such as regulation of blood lipids metabolism [41].

Arterial hypertension is generally associated to dyslipidemia [42]. In this study, L-NAME induced an increase in blood total cholesterol, triglycerides, LDL-cholesterol, and atherogenic index and a decrease in HDL-cholesterol. These modifications were reversed with treatment of rats with MMVC. Most studies have shown that the blockade of NO synthase by L-NAME could lead to an alteration of lipids metabolism [42, 43]. In fact, the nitric oxide plays an important role in the regulation of lipid metabolism. The endothelial and inducible NOS have been shown to be present in the adipose tissue. Endothelial and inducible NOS have been shown to be present in the adipose tissue suggesting its ability to produce NO [44]. NO induces an activation of hepatic sterol regulatory element-binding protein (SREBP)-2, a transcriptional factor necessary for cholesterol metabolism and expression of LDL receptor, resulting in the improvement of the uptake of cholesterol into the hepatic cells. This plays an important role in the normalization of blood cholesterol. So, the inhibition of nitric oxide synthase by L-NAME lead to the rise of total cholesterol, low density lipoprotein (LDL-C), and a low level of high density lipoprotein (HDL-C) [45, 46].

The reduction of blood lipids is an efficient method to prevent and treat cardiovascular affections [47]. These observations suggest that MMVC would exhibit these effects through their ability to raise NO in hypertensive animals. MMVC could also induce an inhibition of reductase hydroxyl methyl glutanyl CoA (HMG-CoA) leading to reduction of the hepatic synthesis and intestinal absorption of cholesterol. Indeed, the inhibition of HMG-CoA by flavonoids, a compound of our plant extract, has been shown. This effect of MMVC on blood lipids could suggest its beneficial effect against lipid peroxidation and further on oxidative stress [48].

It is well known that arterial hypertension is associated with a rise in reactive oxygen species (ROS) [49, 50]. Several studies have shown alteration of oxidative status in LNHR and an improvement after treatment with an antioxidant substance [51, 52]. An increase in reduced NADPH oxidase activity observed in hypertensive animals is responsible for the rise in ROS [53]. In our work, subacute treatment of animals with L-NAME induced an alteration of oxidative status marked by a rise in malondialdehyde level (MDA), a decrease in NO and reduced glutathione levels, and SOD and catalase activities in some organs (the aorta, heart, liver, and kidneys). MMVC (200 and 400 mg/kg) reversed all these modifications observed in hypertensive rats. Previous studies revealed an *in vitro* antiradical activity of MMVC [13]. Furthermore, its preventive antioxidant properties were shown in rats receiving concomitantly L-NAME [15]. The decrease in NO in hypertensive rats could be due to a reduction of its biodisponibility by increasing superoxide anion generally observed in this pathology [54]. The increase in SOD after treatment of hypertensive rats with MMVC is beneficial in the fact that this enzyme reduces the level of superoxide anion by converting it into water and hydrogen peroxide which will be cleaned in turn by catalase [55]. The decrease in reduced glutathione in hypertensive rats suggests its conversion into oxidized glutathione for the purpose of scavenging the important level of ROS. The increase in MDA in hypertensive rats is a consequence of the decrease in antioxidant defense systems resulting in increase in ROS. ROS reacts with biological substances, mainly polyunsaturated fatty acids of cell membrane to produce instable hydroperoxides responsible for important decrease in membrane fluidity [48]. So, the improvement of antioxidant systems observed after treatment of hypertensive rats with MMVC could be responsible for the decrease in ROS and then the decrease in MDA was observed. Similar results were obtained by Ntchapda et al. [56] who had shown that antihypertensive effect of the aqueous extract of *Adansonia digitata* would be linked to their antioxidant potential. This activity of MMVC could also be attributed to the maslinic acid and flavonoids present in this extract, as their inhibitory effects on lipids peroxidation have already been mentioned [57, 58].

## 5. Conclusion

This work showed that the methanol/methylene chloride extract of *Vitex cienkowskii* (MMVC) is able to cure L-NAME-induced hypertension in rats. All these results confirmed the existence of biological effects whenever this plant is empirically used in the treatment of cardiovascular disorders. This plant should be considered as a new therapeutic tool in the treatment of arterial hypertension associated with NO deficiency.

## Figures and Tables

**Table 1:** Effects of the methanol/methylene chloride extract of *V. cienkowskii* on relative weight of organs.

Organs (g/100 g of body weight)	Distilled water	L-NAME	L-NAME + CAPT	L-NAME + MMVC (200 mg/kg)	L-NAME + MMVC (400 mg/kg)
Kidneys	0.26 ± 0.01	0.29 ± 0.01	0.28 ± 0.01	0.24 ± 0.03	0.29 ± 0.05
Liver	2.49 ± 0.10	3.25 ± 0.16^*∗*^	2.85 ± 0.11	2.73 ± 0.23	2.76 ± 0.24
Whole heart	0.29 ± 0.01	0.34 ± 0.00^*∗*^	0.30 ± 0.01	0.30 ± 0.01	0.31 ± 0.02
Left ventricle	0.24 ± 0.01	0.29 ± 0.01^*∗*^	0.25 ± 0.01^$^	0.26 ± 0.01^$^	0.26 ± 0.02^$^
Right ventricle	0.05 ± 0.00	0.05 ± 0.00	0.05 ± 0.01	0.05 ± 0.00	0.05 ± 0.00

Each data represent the mean ± SEM; *n* = 5; ^*∗*^*P* < 0.05 significant difference compared to control (rats receiving distilled water); ^$^*P* < 0.05 significant difference compared to hypertensive control (rats receiving L-NAME only); L-NAME + CAPT: rats treated with L-NAME and captopril at the dose of 25 mg/kg; L-NAME + MMVC (200 mg/kg): rats treated with L-NAME and plant extract (MMVC) at the dose of 200 mg/kg; L-NAME + MMVC (400 mg/kg): rats treated with L-NAME and plant extract (MMVC) at the dose of 400 mg/kg.

**Table 2:** Effects of the methanol/methylene chloride extract of *V. cienkowskii* on hepatic and renal markers.

Parameters		Distilled water	L-NAME	L-NAME + CAPT	L-NAME + MMVC (200 mg/kg)	L-NAME + MMVC (400 mg/kg)
ASAT (U/L)	Serum	31.75 ± 3.45	59.25 ± 3.93^*∗∗*^	37.46 ± 5.34^$$^	30.75 ± 4.18^$$^	36.11 ± 1.29^$$^
	Liver	132.00 ± 5.45	178.50 ± 9.72^*∗∗*^	161.21 ± 4.33^*∗*^	160.79 ± 4.64^*∗*^	146.21 ± 5.70^$$^

ALAT (U/L)	Serum	15.89 ± 0.89	26.21 ± 1.35^*∗∗*^	17.71 ± 0.69^$$^	18.14 ± 0.97^$$^	18.07 ± 1.14^$$^
	Liver	68.64 ± 4.95	96.46 ± 1.86^*∗∗*^	77.96 ± 2.46^$$^	76.00 ± 1.13^$$^	75.89 ± 5.09^$$^

Bilirubin (mg/dL)	Serum	1.90 ± 0.18	2.32 ± 0.09	1.97 ± 0.14	1.85 ± 0.14	1.35 ± 0.11^*∗*^
	Liver	7.28 ± 0.25	8.04 ± 0.40	7.50 ± 0.59	7.26 ± 0.35	6.42 ± 0.25^*∗*^

Creatinine (mg/L)	Serum	26.50 ± 2.48	48.00 ± 3.80^*∗∗*^	36.25 ± 3.38^$^	39.50 ± 3.68^*∗*^	34.50 ± 3.80^$^
	Kidneys	20.50 ± 1.51	39.25 ± 2.31^*∗*^	21.75 ± 1.35^$^	21.50 ± 4.32^$^	19.00 ± 1.50^$^

Each data represent the mean ± SEM; *n* = 5; ^*∗*^*P* < 0.05; ^*∗∗*^*P* < 0.01 significant differences compared to control (rats receiving distilled water); ^$^*P* < 0.05; ^$$^*P* < 0.01 significant differences compared to hypertensive control (rats receiving L-NAME only); L-NAME  +  CAPT: rats treated with L-NAME and captopril at the dose of 25 mg/kg; L-NAME  +  MMVC (200 mg/kg): rats treated with L-NAME and plant extract (MMVC) at the dose of 200 mg/kg; L-NAME  +  MMVC (400 mg/kg): rats treated with L-NAME and plant extract (MMVC) at the dose of 400 mg/kg.

**Table 3:** Effects of the methanol/methylene chloride extract of *V. cienkowskii* on lipid profile.

Parameter	Distilled water	L-NAME	L-NAME + CAPT	L-NAME + MMVC (200 mg/kg)	L-NAME + MMVC (400 mg/kg)
T Chol (mg/dL)	110.15 ± 11.22	156.95 ± 14.90^*∗*^	144.99 ± 8.79	128.09 ± 6.03	89.74 ± 6.46^$$^
TG (mg/dL)	155.00 ± 14.20	190.83 ± 10.88^*∗*^	116.25 ± 11.42^$$^	108.33 ± 11.32^*∗*^^$$^	90.83 ± 8.03^*∗∗*^^$$^
HDL Chol (mg/dL)	60.76 ± 2.93	30.50 ± 3.15^*∗∗*^	54.90 ± 7.37^$$^	62.87 ± 4.83^$$^	56.77 ± 2.61^$$^
LDL Chol (mg/dL)	49.38 ± 10.26	126.45 ± 14.40^*∗∗*^	90.09 ± 12.58	65.2 ± 8.02^$$^	32.9 ± 6.05^$$^
AI	0.81 ± 0.17	4.27 ± 0.57^*∗∗∗*^	1.84 ± 0.42^$$$^	1.08 ± 0.19^$$$^	0.59 ± 0.11^$$$^

Each data represent the mean ± SEM; *n* = 5; ^*∗*^*P* < 0.05; ^*∗∗*^*P* < 0.01 significant differences compared to control (rats receiving distilled water); ^*∗∗∗*^*P* < 0.001 significant differences compared to control (rats receiving distilled water); ^$$^*P* < 0.01 significant differences compared to hypertensive control (rats receiving L-NAME only); ^$$$^*Pp* < 0.001 significant differences compared to hypertensive control (rats receiving L-NAME only); L-NAME + CAPT: rats treated with L-NAME and captopril at the dose of 25 mg/kg; L-NAME + MMVC (200 mg/kg): rats treated with L-NAME and plant extract (MMVC) at the dose of 200 mg/kg; L-NAME + MMVC (400 mg/kg): rats treated with L-NAME and plant extract (MMVC) at the dose of 400 mg/kg. T Chol, total cholesterol; TG, triglycerides; AI, atherogenic index.

**Table 4:** Effects of the methanol/methylene chloride extract of *V. cienkowskii* on oxidative stress markers.

Parameter		Distilled water	L-NAME	L-NAME + CAPT	L-NAME + MMVC (200 mg/kg)	L-NAME + MMVC (400 mg/kg)
NO (mmol/mL)	Aorta	159.75 ± 7.40	68.53 ± 8.26 ^*∗∗*^	142.91 ± 3.88^$$^	121.96 ± 4.07^*∗∗*^^$$^	133.95 ± 4.15^$$^
	Heart	135.35 ± 4.45	69.96 ± 7.39 ^*∗∗*^	132.45 ± 9.07^$$^	107.66 ± 9.00∗^$^	118.61 ± 8.39^$$^
	Liver	180.27 ± 4.01	110.31 ± 6.17^*∗∗*^	160.66 ± 3.03^$$^	153.56 ± 5.20^$$^	156.15 ± 4.53^$$^
	Kidneys	180.08 ± 4.05	130.53 ± 9.13^*∗∗*^	177.22 ± 7.27^$$^	158.53 ± 1.55∗^$^	164.20 ± 4.40^$$^

GSH (mmol/mg of tissue)	Aorta	12.40 ± 0.14	9.78 ± 0.96^*∗∗*^	12.65 ± 0.04^$$^	12.66 ± 0.07^$$^	12.54 ± .0.05^$$^
	Heart	13.45 ± 0.07	10.75 ± 0.71^*∗∗*^	13.24 ± 0.12^$$^	13.74 ± 0.23^$$^	13.65 ± 0.23^$$^
	Liver	14.24 ± 0.24	11.60 ± 0.35^*∗∗*^	13.14 ± 0.13^$$^	13.73 ± 0.07^$$^	13.87 ± 0.21^$$^
	Kidneys	14.73 ± 0.42	12.53 ± 0.32^*∗∗*^	14.04 ± 0.31^$$^	14.24 ± 0.18^$$^	14.32 ± 0.22^$$^

MDA (nmol/mg of proteins)	Aorta	22.14 ± 3.64	48.45 ± 4.02^*∗∗*^	25.13 ± 4.78^$$^	18.18 ± 3.34^$$^	12.10 ± 3.64^$$^
	Heart	65.42 ± 7.86	98.66 ± 6.72^*∗∗*^	66.24 ± 5.74^$^	75.08 ± 4.84^$^	73.06 ± 7.86^$^
	Liver	18.24 ± 1.90	26.63 ± 3.40^*∗*^	20.33 ± 5.01	19.90 ± 1.86	18.55 ± 1.90
	Kidneys	26.51 ± 4.98	46.74 ± 8.10^*∗*^	38.84 ± 2.40	43.87 ± 3.50	38.17 ± 4.98

CAT (H_2_O_2_ destroyed (*µ*M)/min/mg of proteins)	Aorta	10.98 ± 0.61	4.48 ± 0.26^*∗*^	5.83 ± 0.49	8.37 ± 0.97^$^	9.17 ± 0.94^$^
	Heart	12.96 ± 0.81	5.26 ± 0.52^*∗*^	10.10 ± 0.71^$^	9.89 ± 0.52^$^	11.23 ± 1.37^$^
	Liver	10.87 ± 1.28	3.09 ± 0.34^*∗∗*^	7.63 ± 0.70^*∗*^^$$^	5.20 ± 0.61^*∗∗*^	5.62 ± 0.52^*∗∗*^
	Kidneys	6.84 ± 0.67	4.99 ± 0.73^*∗*^	6.19 ± 0.68	7.28 ± 0.38	6.60 ± 0.70

SOD (U/mg of proteins)	Aorta	1.58 ± 0.21	0.56 ± 0.20^*∗*^	1.11 ± 0.31^$^	1.49 ± 0.26^$^	1.04 ± 0.38^$^
	Heart	1.69 ± 0.25	0.91 ± 0.25^*∗*^	0.80 ± 0.13^*∗*^	1.47 ± 0.31^$^	1.40 ± 0.27^$^
	Liver	1.91 ± 0.15	0.96 ± 0.32^*∗*^	1.4 ± 0.42	1.67 ± 0.26^$^	1.62 ± 0.51^$^
	Kidneys	1.73 ± 0.27	0.80 ± 0.24^*∗*^	1.13 ± 0.34	1.24 ± 0.24	1.60 ± 0.23^$^

Each data represent the mean ± SEM; *n* = 5; ^*∗*^*P* < 0.05; ^*∗∗*^*P* < 0.01 significant differences compared to control (rats receiving distilled water); ^$^*Pp* < 0.05; ^$$^*P* < 0.01 significant differences compared to hypertensive control (rats receiving L-NAME only); L-NAME + CAPT: rats treated with L-NAME and captopril at the dose of 25 mg/kg; L-NAME + MMVC (200 mg/kg): rats treated with L-NAME and plant extract (MMVC) at the dose of 200 mg/kg; L-NAME + MMVC (400 mg/kg): rats treated with L-NAME and plant extract (MMVC) at the dose of 400 mg/kg; NO, nitrite oxide; GSH, reduced glutathione; MDA, malondialdehyde; CAT, catalase; SOD, superoxide dismutase.

## Data Availability

The raw data supporting the conclusions of this article will be made available by authors, without under reservation to any qualified scientists.
